# 1-[(Dimethyl­amino)(phen­yl)meth­yl]naphthalen-2-ol

**DOI:** 10.1107/S1600536808027967

**Published:** 2008-09-06

**Authors:** Wenxiang Wang, Hong Zhao

**Affiliations:** a Ordered Matter Science Research Center, College of Chemistry and Chemical Engineering, Southeast University, Nanjing 210096, People’s Republic of China

## Abstract

In the title compound, C_19_H_19_NO, the dihedral angle between the naphthyl ring system and the phenyl ring is 79.83 (6)°. An intra­molecular O—H⋯N hydrogen bond, together with van der Waals inter­actions, stabilizes the mol­ecular conformation.

## Related literature

For related literature, see: Szatmari & Fulop (2004 ▶); Zhao & Sun (2005 ▶).
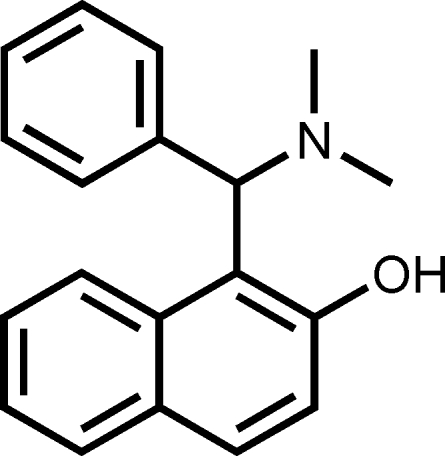


## Experimental

### 

#### Crystal data

C_19_H_19_NO
*M* *_r_* = 277.35
Monoclinic, 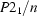

*a* = 9.3297 (10) Å
*b* = 9.2042 (10) Å
*c* = 18.072 (2) Å
β = 103.66 (2)°
*V* = 1508.0 (3) Å^3^
*Z* = 4
Mo *K*α radiation
μ = 0.07 mm^−1^
*T* = 293 (2) K
0.20 × 0.20 × 0.20 mm

#### Data collection

Rigaku SCXmini diffractometer
Absorption correction: multi-scan (*CrystalClear*; Rigaku, 2005 ▶) *T*_min_ = 0.934, *T*_max_ = 0.992
14941 measured reflections
3440 independent reflections
1835 reflections with *I* > 2σ(*I*)
*R*_int_ = 0.083

#### Refinement

*R*[*F* ^2^ > 2σ(*F* ^2^)] = 0.065
*wR*(*F* ^2^) = 0.151
*S* = 0.99
3440 reflections
193 parameters
H-atom parameters constrained
Δρ_max_ = 0.13 e Å^−3^
Δρ_min_ = −0.18 e Å^−3^

### 

Data collection: *CrystalClear* (Rigaku, 2005 ▶); cell refinement: *CrystalClear*; data reduction: *CrystalClear*; program(s) used to solve structure: *SHELXS97* (Sheldrick, 2008 ▶); program(s) used to refine structure: *SHELXL97* (Sheldrick, 2008 ▶); molecular graphics: *SHELXTL* (Sheldrick, 2008 ▶); software used to prepare material for publication: *SHELXTL*.

## Supplementary Material

Crystal structure: contains datablocks I, global. DOI: 10.1107/S1600536808027967/bx2176sup1.cif

Structure factors: contains datablocks I. DOI: 10.1107/S1600536808027967/bx2176Isup2.hkl

Additional supplementary materials:  crystallographic information; 3D view; checkCIF report

## Figures and Tables

**Table 1:** Hydrogen-bond geometry (Å, °)

*D*—H⋯*A*	*D*—H	H⋯*A*	*D*⋯*A*	*D*—H⋯*A*
O1—H1*A*⋯N1	0.82	1.87	2.593 (3)	147

**Fig. 1.**
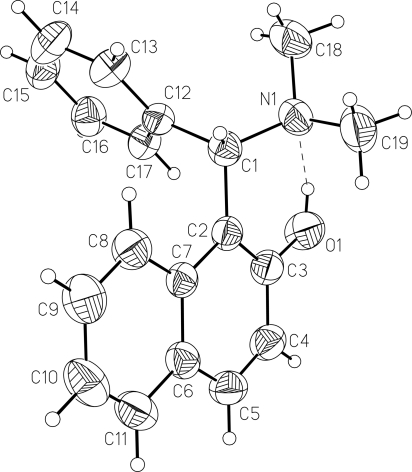
The structure of (I), showing the atom-numbering scheme. Displacement ellipsoids are drawn at the 50% probability level. The intramolecular hydrogen bond is indicated by a dashed line.

## supplementary crystallographic information

### Comment

Compounds derived from naphthalen-2-ol have been of great interest in organic chemistry (Szatmari & Fulop, 2004; Zhao & Sun, 2005). We report herein the crystal structure of the title compound (Fig. 1). The dihedral angle between the naphthyl ring and phenyl ring is 79.83 (6)°. Strong intramolecular O—H···N hydrogen bond [O1—H1A = 0.82 Å, H1A···N1 = 1.87 Å, O1···N1 = 2.593 (3) Å, O1—H1A···N1 = 147°] together with van der Waals interactions stabilize the molecular conformation.

### Experimental

A dry 50 ml flask was charged with benzaldehyde (10 mmol), naphthalen-2-ol (10 mmol), dimethylamine (10 mmol) (33% aq). The mixture was stirred at 100°C for 10 h and then added ethanol (15 ml), after heated under reflux for 30 minutes, the precipitate was filtrated out and washed with ethanol for 2–3 times and purified by recrystallization from dichloromethane to give the target material.

### Refinement

All the hydrogen atoms were calculated geometrically and with C—H distances ranging from 0.93 to 0.98 Å. C_aryl_—H = 0.93 Å, with *U*_iso_(H) = 1.2*U*_eq_(C). C_methyl_—H = 0.96 Å, with *U*_iso_(H) = 1.5*U*_eq_(C). O—H = 0.82 Å with *U*_iso_(H) = 1.5*U*_eq_(O).

### Crystal data

**Table:**

C_19_H_19_NO	*F*(000) = 592
*M**_r_* = 277.35	*D*_x_ = 1.222 Mg m^−^^3^
Monoclinic, *P*2_1_/*n*	Mo *K*α radiation, λ = 0.71073 Å
Hall symbol: -P 2yn	Cell parameters from 2352 reflections
*a* = 9.3297 (10) Å	θ = 2.8–27.5°
*b* = 9.2042 (10) Å	µ = 0.08 mm^−^^1^
*c* = 18.072 (2) Å	*T* = 293 K
β = 103.66 (2)°	Prism, colourless
*V* = 1508.0 (3) Å^3^	0.20 × 0.20 × 0.20 mm
*Z* = 4	

### Data collection

**Table:**

Rigaku SCXmini diffractometer	3440 independent reflections
Radiation source: fine-focus sealed tube	1835 reflections with *I* > 2σ(*I*)
graphite	*R*_int_ = 0.083
Detector resolution: 13.6612 pixels mm^-1^	θ_max_ = 27.5°, θ_min_ = 3.2°
ω scans	*h* = −11→12
Absorption correction: multi-scan (CrystalClear; Rigaku, 2005)	*k* = −11→11
*T*_min_ = 0.934, *T*_max_ = 0.992	*l* = −23→23
14941 measured reflections	

### Refinement

**Table:**

Refinement on *F*^2^	Primary atom site location: structure-invariant direct methods
Least-squares matrix: full	Secondary atom site location: difference Fourier map
*R*[*F*^2^ > 2σ(*F*^2^)] = 0.065	Hydrogen site location: inferred from neighbouring sites
*wR*(*F*^2^) = 0.151	H-atom parameters constrained
*S* = 0.99	*w* = 1/[σ^2^(*F*_o_^2^) + (0.061*P*)^2^] where *P* = (*F*_o_^2^ + 2*F*_c_^2^)/3
3440 reflections	(Δ/σ)_max_ < 0.001
193 parameters	Δρ_max_ = 0.13 e Å^−^^3^
0 restraints	Δρ_min_ = −0.18 e Å^−^^3^

### Special details

**Table:**

Geometry. All e.s.d.'s (except the e.s.d. in the dihedral angle between two l.s. planes) are estimated using the full covariance matrix. The cell e.s.d.'s are taken into account individually in the estimation of e.s.d.'s in distances, angles and torsion angles; correlations between e.s.d.'s in cell parameters are only used when they are defined by crystal symmetry. An approximate (isotropic) treatment of cell e.s.d.'s is used for estimating e.s.d.'s involving l.s. planes.
Refinement. Refinement of *F*^2^ against ALL reflections. The weighted *R*-factor *wR* and goodness of fit *S* are based on *F*^2^, conventional *R*-factors *R* are based on *F*, with *F* set to zero for negative *F*^2^. The threshold expression of *F*^2^ > σ(*F*^2^) is used only for calculating *R*-factors(gt) *etc*. and is not relevant to the choice of reflections for refinement. *R*-factors based on *F*^2^ are statistically about twice as large as those based on *F*, and *R*- factors based on ALL data will be even larger.

### Fractional atomic coordinates and isotropic or equivalent isotropic displacement parameters (Å^2^)

**Table:**

	*x*	*y*	*z*	*U*_iso_*/*U*_eq_	
C1	0.9601 (2)	0.1887 (2)	0.19972 (12)	0.0402 (5)	
H1	1.0000	0.2728	0.2312	0.048*	
C2	0.8401 (2)	0.2439 (2)	0.13328 (12)	0.0376 (5)	
C3	0.7170 (2)	0.1608 (2)	0.10134 (13)	0.0431 (5)	
C4	0.6082 (2)	0.2123 (3)	0.03982 (13)	0.0486 (6)	
H4	0.5253	0.1558	0.0202	0.058*	
C5	0.6229 (3)	0.3438 (3)	0.00864 (12)	0.0490 (6)	
H5	0.5498	0.3759	−0.0325	0.059*	
C6	0.7468 (2)	0.4334 (2)	0.03727 (12)	0.0428 (5)	
C7	0.8563 (2)	0.3830 (2)	0.09998 (12)	0.0379 (5)	
C8	0.9798 (3)	0.4747 (2)	0.12697 (13)	0.0489 (6)	
H8	1.0547	0.4442	0.1677	0.059*	
C9	0.9911 (3)	0.6069 (3)	0.09435 (15)	0.0597 (7)	
H9	1.0730	0.6651	0.1135	0.072*	
C10	0.8826 (3)	0.6557 (3)	0.03331 (15)	0.0624 (7)	
H10	0.8914	0.7458	0.0116	0.075*	
C11	0.7632 (3)	0.5708 (3)	0.00549 (14)	0.0542 (6)	
H11	0.6904	0.6038	−0.0355	0.065*	
C12	1.0864 (2)	0.1206 (2)	0.17158 (12)	0.0396 (5)	
C13	1.2301 (2)	0.1639 (3)	0.20272 (14)	0.0540 (6)	
H13	1.2489	0.2341	0.2408	0.065*	
C14	1.3465 (3)	0.1033 (3)	0.17760 (16)	0.0653 (8)	
H14	1.4427	0.1328	0.1992	0.078*	
C15	1.3208 (3)	0.0005 (3)	0.12133 (16)	0.0593 (7)	
H15	1.3990	−0.0397	0.1046	0.071*	
C16	1.1786 (3)	−0.0430 (3)	0.08969 (14)	0.0540 (6)	
H16	1.1604	−0.1129	0.0515	0.065*	
C17	1.0626 (2)	0.0171 (2)	0.11468 (13)	0.0462 (6)	
H17	0.9667	−0.0128	0.0928	0.055*	
C18	1.0071 (3)	0.0025 (3)	0.30073 (15)	0.0662 (8)	
H18A	1.0796	0.0664	0.3305	0.099*	
H18B	1.0540	−0.0629	0.2724	0.099*	
H18C	0.9609	−0.0523	0.3339	0.099*	
C19	0.8097 (3)	0.1744 (3)	0.29155 (15)	0.0677 (8)	
H19A	0.7613	0.1103	0.3198	0.102*	
H19B	0.7372	0.2311	0.2569	0.102*	
H19C	0.8749	0.2378	0.3261	0.102*	
N1	0.8956 (2)	0.0879 (2)	0.24811 (10)	0.0481 (5)	
O1	0.69398 (18)	0.02542 (17)	0.12688 (10)	0.0592 (5)	
H1A	0.7539	0.0097	0.1672	0.089*	

### Atomic displacement parameters (Å^2^)

**Table:**

	*U*^11^	*U*^22^	*U*^33^	*U*^12^	*U*^13^	*U*^23^
C1	0.0412 (12)	0.0364 (12)	0.0438 (13)	−0.0011 (10)	0.0117 (10)	0.0019 (10)
C2	0.0360 (12)	0.0381 (12)	0.0410 (13)	0.0018 (10)	0.0135 (10)	0.0014 (9)
C3	0.0402 (13)	0.0405 (12)	0.0503 (15)	0.0013 (11)	0.0141 (11)	0.0035 (10)
C4	0.0397 (13)	0.0556 (15)	0.0491 (15)	−0.0006 (11)	0.0073 (11)	−0.0021 (12)
C5	0.0462 (14)	0.0590 (15)	0.0405 (14)	0.0094 (12)	0.0073 (11)	0.0010 (11)
C6	0.0464 (13)	0.0427 (13)	0.0423 (13)	0.0039 (11)	0.0166 (11)	0.0019 (10)
C7	0.0370 (12)	0.0399 (12)	0.0397 (13)	0.0043 (10)	0.0148 (10)	0.0011 (10)
C8	0.0486 (14)	0.0448 (13)	0.0536 (15)	−0.0018 (11)	0.0125 (11)	0.0029 (11)
C9	0.0646 (17)	0.0475 (15)	0.0683 (18)	−0.0114 (13)	0.0183 (14)	0.0011 (13)
C10	0.085 (2)	0.0431 (14)	0.0640 (18)	−0.0012 (14)	0.0260 (16)	0.0130 (13)
C11	0.0666 (17)	0.0503 (15)	0.0475 (15)	0.0100 (13)	0.0168 (13)	0.0092 (12)
C12	0.0361 (12)	0.0408 (12)	0.0418 (13)	0.0009 (10)	0.0086 (10)	0.0067 (10)
C13	0.0412 (14)	0.0561 (15)	0.0619 (16)	−0.0012 (12)	0.0065 (12)	−0.0048 (12)
C14	0.0363 (13)	0.0688 (18)	0.089 (2)	−0.0012 (13)	0.0103 (13)	−0.0003 (16)
C15	0.0493 (15)	0.0571 (16)	0.0778 (19)	0.0072 (13)	0.0276 (14)	0.0082 (14)
C16	0.0569 (16)	0.0489 (14)	0.0606 (16)	0.0015 (12)	0.0223 (13)	−0.0007 (12)
C17	0.0400 (13)	0.0470 (13)	0.0521 (15)	−0.0034 (11)	0.0122 (11)	−0.0014 (11)
C18	0.0735 (18)	0.0670 (17)	0.0590 (17)	0.0127 (15)	0.0176 (14)	0.0236 (14)
C19	0.0742 (19)	0.0733 (19)	0.0667 (18)	0.0143 (15)	0.0389 (15)	0.0139 (14)
N1	0.0520 (12)	0.0487 (12)	0.0467 (11)	0.0042 (9)	0.0175 (9)	0.0115 (9)
O1	0.0501 (10)	0.0480 (10)	0.0767 (13)	−0.0078 (8)	0.0093 (9)	0.0119 (9)

### Geometric parameters (Å, °)

**Table:**

C1—N1	1.496 (3)	C11—H11	0.9300
C1—C2	1.523 (3)	C12—C17	1.381 (3)
C1—C12	1.524 (3)	C12—C13	1.385 (3)
C1—H1	0.9800	C13—C14	1.389 (3)
C2—C3	1.387 (3)	C13—H13	0.9300
C2—C7	1.438 (3)	C14—C15	1.368 (4)
C3—O1	1.364 (2)	C14—H14	0.9300
C3—C4	1.399 (3)	C15—C16	1.374 (3)
C4—C5	1.356 (3)	C15—H15	0.9300
C4—H4	0.9300	C16—C17	1.383 (3)
C5—C6	1.415 (3)	C16—H16	0.9300
C5—H5	0.9300	C17—H17	0.9300
C6—C11	1.412 (3)	C18—N1	1.462 (3)
C6—C7	1.413 (3)	C18—H18A	0.9600
C7—C8	1.419 (3)	C18—H18B	0.9600
C8—C9	1.367 (3)	C18—H18C	0.9600
C8—H8	0.9300	C19—N1	1.480 (3)
C9—C10	1.384 (3)	C19—H19A	0.9600
C9—H9	0.9300	C19—H19B	0.9600
C10—C11	1.356 (3)	C19—H19C	0.9600
C10—H10	0.9300	O1—H1A	0.8200
			
N1—C1—C2	110.19 (17)	C6—C11—H11	119.2
N1—C1—C12	112.93 (17)	C17—C12—C13	118.1 (2)
C2—C1—C12	110.88 (17)	C17—C12—C1	122.09 (19)
N1—C1—H1	107.5	C13—C12—C1	119.8 (2)
C2—C1—H1	107.5	C12—C13—C14	120.6 (2)
C12—C1—H1	107.5	C12—C13—H13	119.7
C3—C2—C7	118.36 (19)	C14—C13—H13	119.7
C3—C2—C1	121.80 (18)	C15—C14—C13	120.5 (2)
C7—C2—C1	119.78 (18)	C15—C14—H14	119.8
O1—C3—C2	123.0 (2)	C13—C14—H14	119.8
O1—C3—C4	115.8 (2)	C14—C15—C16	119.6 (2)
C2—C3—C4	121.2 (2)	C14—C15—H15	120.2
C5—C4—C3	120.5 (2)	C16—C15—H15	120.2
C5—C4—H4	119.8	C15—C16—C17	120.0 (2)
C3—C4—H4	119.8	C15—C16—H16	120.0
C4—C5—C6	121.5 (2)	C17—C16—H16	120.0
C4—C5—H5	119.3	C12—C17—C16	121.2 (2)
C6—C5—H5	119.3	C12—C17—H17	119.4
C11—C6—C7	119.5 (2)	C16—C17—H17	119.4
C11—C6—C5	122.1 (2)	N1—C18—H18A	109.5
C7—C6—C5	118.4 (2)	N1—C18—H18B	109.5
C6—C7—C8	117.06 (19)	H18A—C18—H18B	109.5
C6—C7—C2	120.06 (19)	N1—C18—H18C	109.5
C8—C7—C2	122.9 (2)	H18A—C18—H18C	109.5
C9—C8—C7	121.4 (2)	H18B—C18—H18C	109.5
C9—C8—H8	119.3	N1—C19—H19A	109.5
C7—C8—H8	119.3	N1—C19—H19B	109.5
C8—C9—C10	121.1 (2)	H19A—C19—H19B	109.5
C8—C9—H9	119.5	N1—C19—H19C	109.5
C10—C9—H9	119.5	H19A—C19—H19C	109.5
C11—C10—C9	119.4 (2)	H19B—C19—H19C	109.5
C11—C10—H10	120.3	C18—N1—C19	109.61 (19)
C9—C10—H10	120.3	C18—N1—C1	113.02 (18)
C10—C11—C6	121.6 (2)	C19—N1—C1	108.65 (18)
C10—C11—H11	119.2	C3—O1—H1A	109.5

### Hydrogen-bond geometry (Å, °)

**Table:**

*D*—H···*A*	*D*—H	H···*A*	*D*···*A*	*D*—H···*A*
O1—H1A···N1	0.82	1.87	2.593 (3)	147

## References

[bb1] Rigaku (2005). *CrystalClear* Rigaku Corporation, Tokyo, Japan.

[bb2] Sheldrick, G. M. (2008). *Acta Cryst.* A**64**, 112–122. 10.1107/S0108767307043930 18156677

[bb3] Szatmari, I. & Fulop, F. (2004). *Curr. Org. Synth.* **1**, 155-165.

[bb4] Zhao, B. & Sun, Y.-X. (2005). *Acta Cryst.* E**61**, m652–m653.

